# Estimating transfection efficiency in differentiated and undifferentiated neural cells

**DOI:** 10.1186/s13104-019-4249-5

**Published:** 2019-04-15

**Authors:** Abeer A. Alabdullah, Basma Al-Abdulaziz, Hanan Alsalem, Amna Magrashi, Subramanian M. Pulicat, Amer A. Almzroua, Falah Almohanna, Abdullah Mohamed Assiri, Nada A. Al Tassan, Bashayer R. Al-Mubarak

**Affiliations:** 1 Behavioral Genetics Unit, Department of Genetics, King Faisal Specialist Hospital and Research Center, Riyadh, 11211 Saudi Arabia; 2 National Center for Genomics Technology, King Abdulaziz City for Science and Technology, Riyadh, Saudi Arabia; 3 Stem Cell & Tissue Re-Engineering Program, King Faisal Specialist Hospital and Research Center, Riyadh, 11211 Saudi Arabia; 4 Department of Comparative Medicine, King Faisal Specialist Hospital and Research Center, Riyadh, 11211 Saudi Arabia; 5 College of Medicine, Alfaisal University, Riyadh, Saudi Arabia; 6 Institute of Research and Medical Consultations, Imam Abdulrahman Bin Faisal University, Dammam, Saudi Arabia; 7 Clinical Laboratory Department, College of Applied Medical Sciences, King Saud University, Riyadh, Saudi Arabia

**Keywords:** Neuroblastoma cell lines, Primary cortical neurons, Primary cortical astrocytes, Lipofection, Transfection efficiency

## Abstract

**Objective:**

Delivery of constructs for silencing or over-expressing genes or their modified versions is a crucial step for studying neuronal cell biology. Therefore, efficient transfection is important for the success of these experimental techniques especially in post-mitotic cells like neurons. In this study, we have assessed the transfection rate, using a previously established protocol, in both primary cortical cultures and neuroblastoma cell lines. Transfection efficiencies in these preparations have not been systematically determined before.

**Results:**

Transfection efficiencies obtained herein were (10–12%) for neuroblastoma, (5–12%) for primary astrocytes and (1.3–6%) for primary neurons. We also report on cell-type specific transfection efficiency of neurons and astrocytes within primary cortical cultures when applying cell-type selective transfection protocols. Previous estimations described in primary cortical or hippocampal cultures were either based on general observations or on data derived from unspecified number of biological and/or technical replicates. Also to the best of our knowledge, transfection efficiency of pure primary neuronal cultures or astrocytes cultured in the context of pure or mixed (neurons/astrocytes) population cultures have not been previously determined. The transfection strategy used herein represents a convenient, and a straightforward tool for targeted cell transfection that can be utilized in a variety of in vitro applications.

**Electronic supplementary material:**

The online version of this article (10.1186/s13104-019-4249-5) contains supplementary material, which is available to authorized users.

## Introduction

Successful delivery of plasmid DNA or short/small interfering RNA (RNAi) is a crucial step for studying neuronal cell biology at a molecular level, through silencing and over-expression of wild type or mutant versions of a gene. Therefore, efficient transfection is important for the success of these experimental techniques especially in “challenging” post-mitotic cells like neurons. Various methods have been developed for introducing exogenous constructs into primary neurons such as electroporation (nucleofection), calcium phosphate, viral vectors and magnetofection [1, 2]. Each method has its own advantages and limitations. For instance, calcium phosphate is very inexpensive and easy to perform; but it has low transfection efficiency and is hard to reproduce being sensitive to pH, temperature and incubation time [3, 4]. On the other hand, transfection efficiencies as high as 85–95% were reported using nucleofection, however, this method has a number of limitations, mainly the need for special equipment and the immediate transfection of neurons after isolation [5]. Similarly, high gene delivery rates have been achieved with viral vectors in primary neurons, but these vectors have a number of drawbacks; which include, special biosafety requirements, gene integration, limitations on the insert size, high-cost and labor-intensive [2]. As for magnetofection, transfection rates of > 45% were documented for this method in primary motor neurons; however, this method requires special equipment and gives optimal results only with young (DIV2) neurons [1].

Among the plethora of gene delivery methods, lipofection is considered the “gold-standard” to which other techniques are usually benchmarked. This is mainly due to its ability to efficiently introduce nucleic acids (DNA and RNAi) into a broad range of cell types, even difficult-to-transfect primary mammalian cells, using simple protocols that have shown high reproducibility and comparatively low toxicity [2, 4]. The continuing popularity of this method is reflected by the number of citing publications that have been on the rise since its launch in 1999. A quick Google scholar search returns over 51,200 articles for the term “lipofectamine” and 26,000 for the term “lipofectamine + neurons” (search conducted on 26th March 19).

Lipofection efficiency has been previously assessed in primary cortical and hippocampal cultures [6, 7], however, these cultures are most likely mixed neuronal-glial preparations in which nor the proportion of each cell type, neither cell type-specific transfection efficiency was determined. Moreover, the reported estimations in primary cortical or hippocampal cultures were either based on general observations (no data was provided) or on data derived from unspecified number of biological and/or technical replicates. Here we present a quantitative evaluation of transfection rates in various neural preparations using Lipofectamine 2000^®^. This commercial formulation is widely used for gene delivery in fetal and neonatal primary cells [4, 8–10]. In this study, we used a previously established transient transfection protocol [11] and determined the transfection efficiency in 5 different types of rodent neural cultures.

## Main text

### Materials and methods

#### Preparation of primary cortical cultures and neuroblastoma cell lines

Animal handling and culling was carried out in accordance with schedule 1 of the standard guidelines on human killing of animals, whereby pregnant dams were euthanized by a trained personnel using CO2, while fetuses were euthanized by decapitation with surgical scissors. Cortical neurons were cultured from embryonic day 17.5 or 18.5 (E17.5/18.5) CD1 or C57BL/6 mouse pups as previously described [12]. Dissociated cortices were plated at a density of 13 × 10^4^ cells/cm^2^ in a 24-well plate. Three types of cortical cultures were generated: astrocyte-containing (AC), astrocyte-free (AF) and astrocytes enriched cultures (AE). Undifferentiated rat B35 and B104 neuroblastoma cells were routinely grown in 25 cm^2^ flasks and subcultured when confluent. Detailed information is provided in Additional file 1.

#### Transient transfection and plasmids

Primary cortical cultures (on DIV7/8) or neuroblastoma cells (B35 and B104) were transiently transfected using Lipofectamine 2000 (Invitrogen) and pEGFP-N3 (Clenotech) following a previously described protocol [11]. Primary cortical cells were transferred to serum free non-trophic medium 2–4 h prior to transfection. A DNA (µg): Lipofectamine (µl) ratio of (1:3.88) was applied to each well. For AE cultures and neuroblastoma cell lines, the same protocol was applied to cells at a confluency of 60–80% with two modifications: (1) cells were seeded in 6-well plates, (2) the recommended transfection medium was replaced with Opti-MEM™ I Reduced Serum Medium (Gibco, France). Neuroblastoma cell lines and AE cultures were transfected with pEGFP-N3:Lipofectamine ratio of (3 µg:11.65 µl/well). Whereas, AC and AF cultures were grown in 24-well plates and transfected with pEGFP-N3:Lipofectamine ratio of (0.6 µg:2.33 µl/well). At the end of the transfection period, the medium on the cells was replaced with either cell-conditioned media (primary cortical cultures) or fresh serum-free growth medium (for neuroblastoma and AE cultures) to prevent cytotoxicity. Finally, cells were fixed either 24 or 48 h post-transfection (hpt), before staining with the desired anti-bodies.

#### Cell viability assessment

Cell viability was determined using FITC Annexin V/Dead Cell Apoptosis Kit with FITC annexin V and PI, for Flow Cytometry (Invitrogen) according to the manufacture’s protocol. A more detailed description of the protocol is provided in Additional file 1.

#### Immunocytochemistry and imaging

Immunofluorescence was performed as previously described [12]. All anti-bodies used in this study are listed in Additional file 1: Table S1. Non-saturated images were acquired using Nikon epi-fluorescent microscope under a 10× objective lens. Image analysis was performed with (Image j) program. For primary cortical cultures phenotyping, we first quantified the total number of cells in each well, by staining the cells with the nuclear marker (DAPI) and excluding ones with pyknotic nuclei. Then percentage of neurons or astrocytes was determined by calculating the percentage of NeuN^+^ and GFAP^+^ cells in the total population (DAPI^+^).

Transfection efficiency in the dividing cells studied here (B35/B104), comprising of a homogenous population of cells, was determined by quantifying the percentage of GFP^+^ cells within the total (DAPI^+^) population. Whereas in primary cortical cultures, the efficiency was determined at a cell-type level using cell identity markers.

### Results and discussion

#### Determining viability of transfected cells

Different gene delivery strategies can have various cytotoxic effects which in turn can confound determination of transfection efficiency. We therefore have assessed toxicity of Lipofectamine-mediated transfection in our preparations. In general low cytotoxicity was observed for all tested neural cultures except AE. Lipofectamine showed the lowest toxicity in neuroblastoma B35 and B104 with cell death increase of only 5.2 and 7.1% from the baseline, respectively (Additional file 1: Fig. S1A, B). Similarly, good survival rates were achieved for AC and AF with only 11 and 12% increase in cytotoxicity levels over baseline, respectively (Additional file 1: Fig. S1C, D). As for AE, relatively high toxicity was observed even in non-transfected cells despite of their normal morphological appearance (Additional file 1: Fig. S2). The overestimation of dead cells may be due to false positive events resulting from PI staining of RNA (see Additional file 1). Considering the above, we applied an alternative method for estimating cytotoxicity in AE using DAPI nuclear staining and measured death by calculating the percentage of pyknotic nuclei in the total population. A modest toxicity was observed with a 17% increase in cell death levels over baseline (Additional file 1: Fig. S1E).

#### Determining transfection efficiency in neuroblastoma cells

Undifferentiated rodent neuroblastoma cell lines such as B35 and B104, are useful in vitro models of CNS for studying important aspects of neurobiology associated with neurodevelopmental processes such as differentiation, neurite outgrowth, cell migration and cell death [13, 14].

**Fig. 1:**
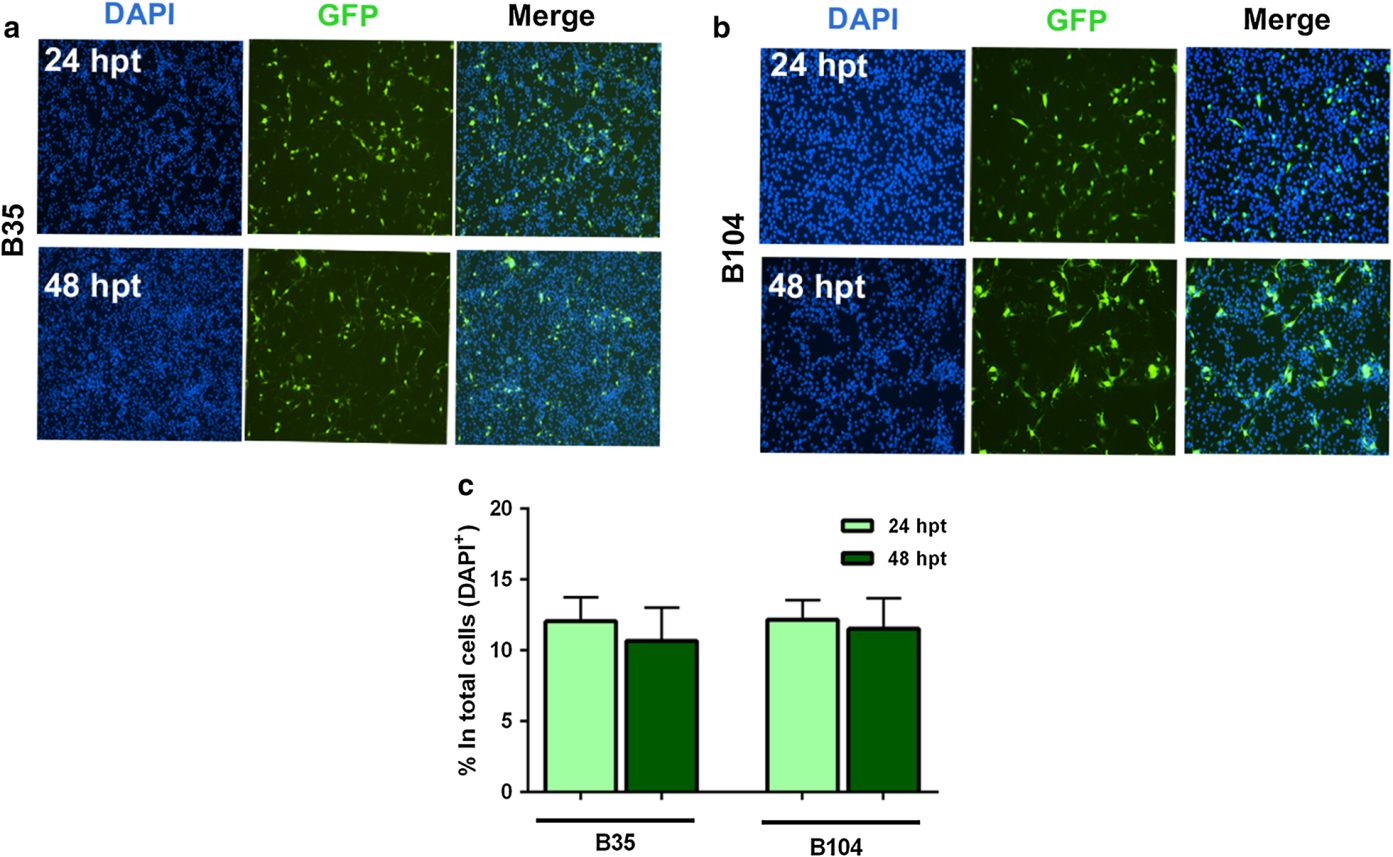
Transfection efficiency in B35 and B104 neuroblastoma cell lines. Representative images of transfected undifferentiated neuroblastoma cell lines. **a** (GFP^+^) B35 cells. **b** (GFP^+^) B104 cells. **c** Quantitative analysis of the percentage of GFP^+^ cells among the total DAPI^+^ cells (total population). For B35, transfection efficiency at 24 hpt was 12.06% SEM ± 0.97 and 10.67% SEM ± 1.34 at 48 hpt. Similar rates were achieved for B104 cells, (mean value 12.2% SEM ± 0.80) at 24 hpt and (mean value 11.5% SEM ± 1.24) at 48 hpt. Cells were transfected at 60–80% confluency in 6-well plates for the indicated periods and about 9–11 fields were imaged per well. Each experiment was done in replicates (2 or 3 wells/condition) from 3 independent cultures. Between (8000 and 24,000 cells) were quantified per experiment. Data represents mean value ± SEM. Nuclear staining DAPI (blue). GFP (green)

Efficiencies were determined at 24 and 48 hpt to allow for high transgene expression [15] (Fig. 1a, b). In B35 cells, efficiencies were comparable for both time points. Moreover, similar rates were achieved for B104 cells (Fig. 1c).

#### Phenotyping of mouse primary cortical cultures

Unlike cell-lines, primary cultures contain heterogeneous population of cells. Therefore, we carried out cell phenotyping at DIV7/8 using the typical neuronal and astrocytic markers (NeuN and GFAP) (Additional file 1: Fig. S3A). The proportion of neurons and astrocytes was calculated as the percentage of NeuN^+^ or GFAP^+^ cells/total cell population (DAPI^+^ nuclei). Dead cells with pyknotic nuclei were excluded.

Our AF cultures were almost completely devoid of astrocytes consisting of 99% neurons, while the AC cultures were composed of 90% neurons and approximately 10% astrocytes. The AE cultures, on the other hand, were predominantly astrocytic containing about 95% GFAP^+^ cells (Additional file 1: Fig. S3B). These findings are consistent with previous studies [16, 17].

#### Determining transfection efficiency in primary astrocytes

The ability to generate different types of cortical cultures with various (neurons vs glia) proportions facilitates the study of these cells in the context of pure or mixed population cultures. This is important when investigating cell-type specific responses or cell–cell interaction.

**Fig. 2:**
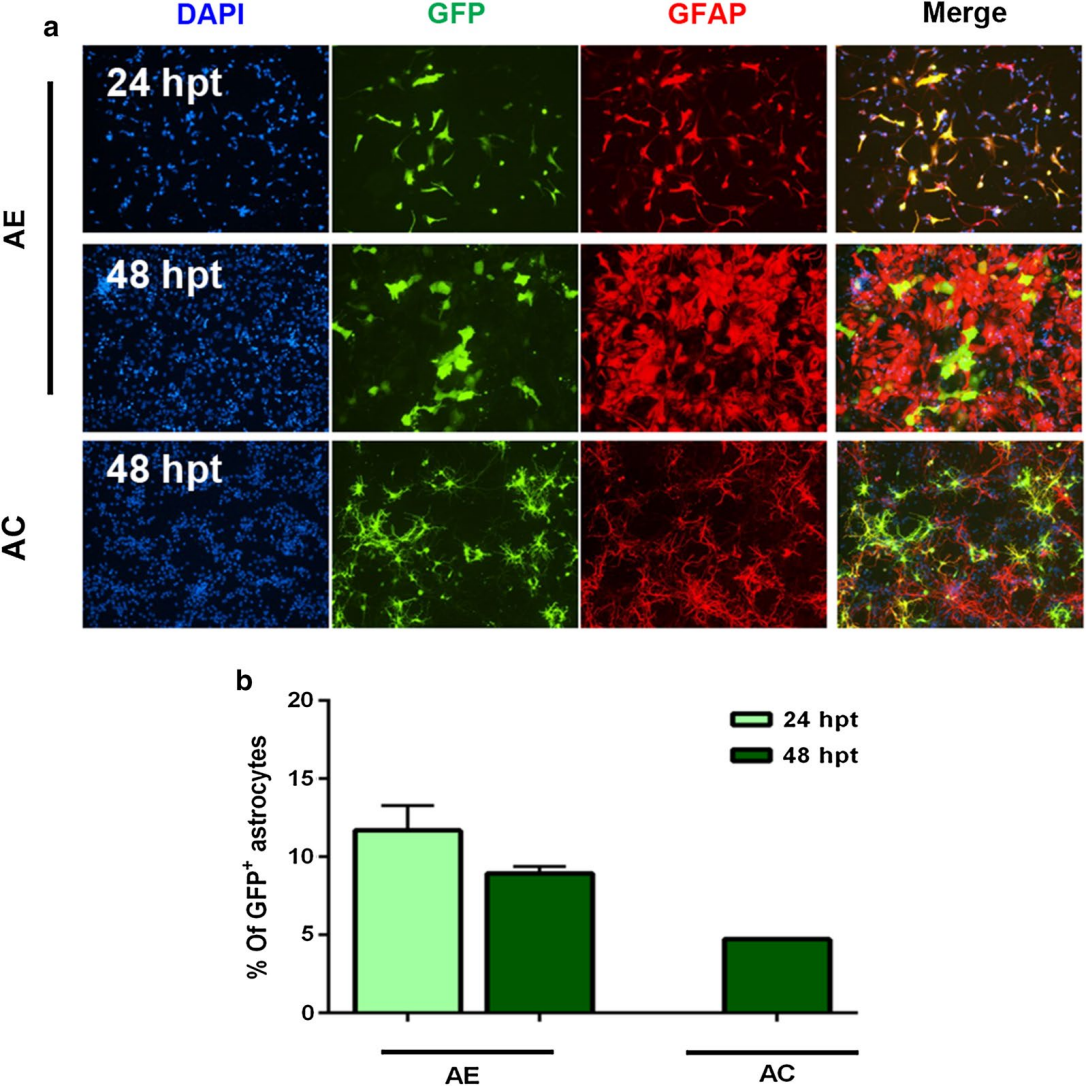
Transfection efficiency of astrocytes in primary cortical cultures. **a** Representative images of transfected astrocytes (GFP^+^/GFAP^+^) in AE and in AC cultures. AE cultures transfected at 60–80% confluency in 6-well plates for the indicated periods and about 10 fields were imaged per well. AC cultures transfected at DIV2 for 48 h in 24-well plates and 7 fields were imaged per well. Each experiment was done in replicates (2 or 3 wells/condition). Total number of cells quantified in each experiment was; (3500–7800 cells) for AC and (2900–7800 cells) for AE. **b** Quantitative analysis of the percentage of GFP^+^/GFAP^+^ cells among the total DAPI^+^ cells (total population, blue). Mean transfection efficiency of astrocytes in AE was obtained from 3 and 2 independent cultures for 24 hpt and 48 hpt experiments, respectively. Mean transfection efficiency of astrocytes in AC was obtained from 2 independent cultures. Data represents mean value ± SEM

Here we have assessed the transfection efficiency in astrocytes when cultured alone (AE cultures) (Fig. 2a). Both tested time points gave comparable transfection efficiencies (Fig. 2b).

To evaluate the transfection efficiency of astrocytes in our AC cultures, we performed transfections on DIV2. Introducing the transfection mixture at this developmental stage have been observed to preferentially transfect astrocytes in this type of cell culture [11]. Assessment of the transfection efficiency 48 hpt, revealed that the GFP^+^ cells were predominantly astrocytes (74%) (Fig. 2a and Additional file 1: Fig. S4A). The overall percentage of transfected cells (total GFP^+^/NeuN^+^ and GFP^+^/GFAP^+^) was 6.4% of which 1.6% were neurons and 4.7% were astrocytes (Fig. 2b).

#### Determining transfection efficiency in primary neurons

We decided to assess the transfection efficiency of neurons at DIV7/8, a developmental stage typically chosen for carrying out transfections in primary neurons [8–10, 12, 18, 19].

**Fig. 3:**
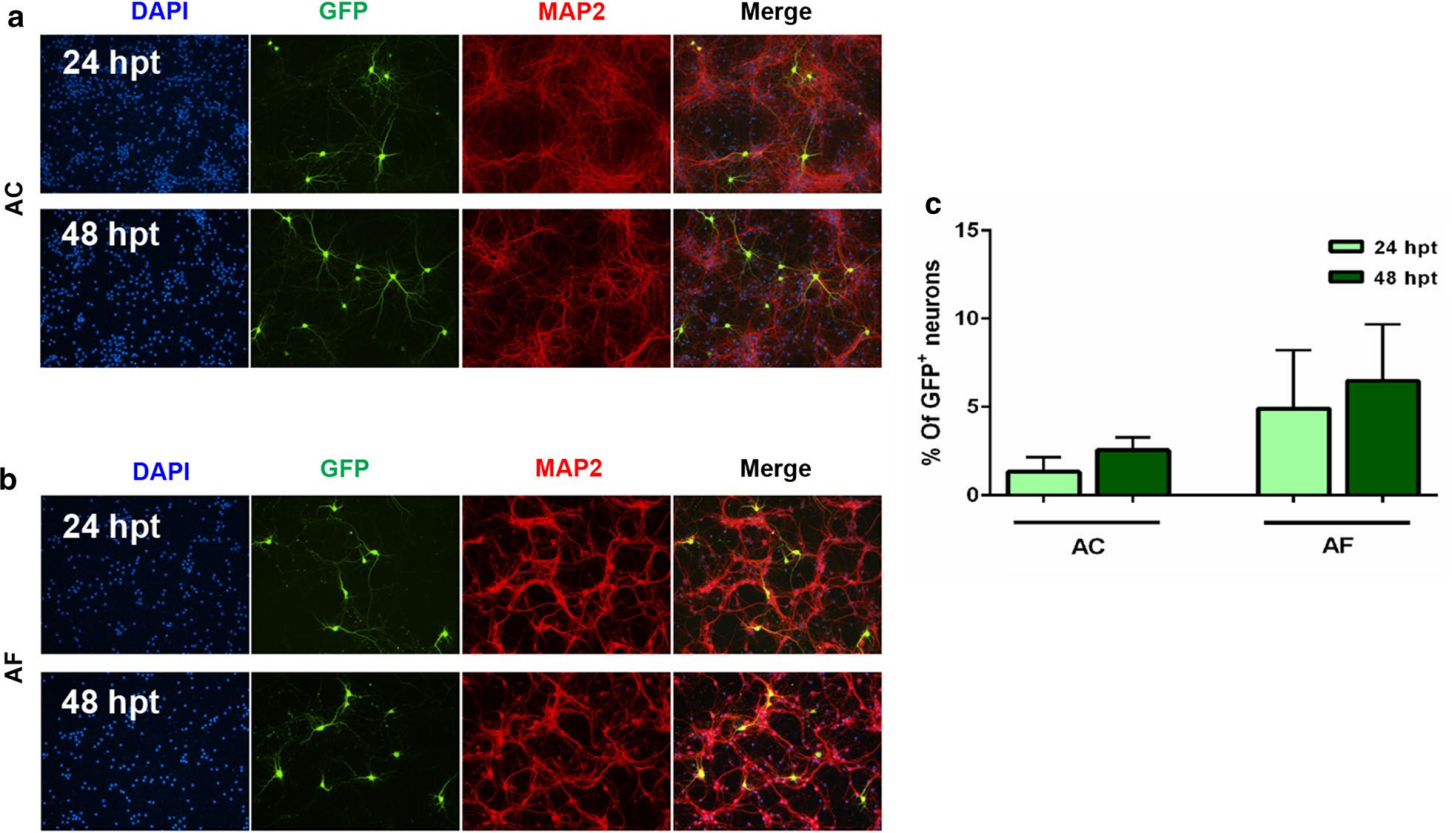
Transfection efficiency of neurons in primary cortical cultures. Representative images of transfected neurons (GFP^+^/MAP2^+^) in AC (**a**) and in AF (**b**) cultures. Cortical cultures were transfected at DIV7/8 in 24-well plates for the indicated periods. **c** Quantitative analysis of the percentage of GFP^+^/MAP2^+^ cells among the total DAPI^+^ cells (total population, blue). The transfection rate for AC was 1.3% ± 0.3 at 24 hpt and 2.6% ± 0.4 at 48 hpt. About 6–9 fields were imaged per well and each experiment was done in triplicates (3 wells/condition). Mean transfection efficiency of neurons in AC was obtained from 7 and 3 independent cultures for 24 hpt and 48 hpt experiments, respectively. About (5164–20,014 cells) were quantified in each experiment for AC. As for the AF transfection rate was 4.9% ± 1.5 at 24 hpt and 6.4% ± 1.9 at 48 hpt. Mean transfection efficiency of neurons in AF was obtained from 5 and 3 independent cultures for 24 hpt and 48 hpt experiments, respectively. About (476–6097 cells) were quantified in each experiment for AF. Data represents mean value ± S.E.M

The protocol applied here reportedly selects neurons over astrocytes [11, 17], and in our AC cultures it almost exclusively transfects neurons (only 3 GFP^+^/GFAP^+^ cells were detected vs 1330 GFP^+^ neurons in a total of 84,685 cells analyzed) (Fig. 3a and Additional file 1: Fig. S4B). The achieved transfection rate was 1.3% and 2.6% at 24 and 48 hpt, respectively (Fig. 3c).

As for the AF (cultures), a higher transfection rate was obtained (4.9% at 24 hpt and 6.4% at 48 hpt) (Fig. 3b, c). To our knowledge, transfection rate has not been previously determined in rodent primary AF “pure neuronal” cultures. However, the achieved transfection efficiencies in our primary neuronal cultures are within the previously reported range (1–5%) using the same method [2, 11, 17].

In an attempt to improve transfection efficiency, we trialed various Lipofectamine:DNA ratios and tested transfecting cells at DIV4 instead of DIV7/8. However, none of the tested conditions gave better results (see Additional file 1).

## Limitations

In this study transfection efficiency was determined by testing a limited range of DNA: Lipofectamine ratio, also other more sensitive methods could be employed to evaluate transfection efficiency such as flow cytometry. Lipofection is a popular gene delivery method due, not only to its high gene transfer efficiency, but also due to other considerations such as ease of use, cost effectiveness, reproducibility and time- and labor-saving. To our knowledge, transfection efficiencies using Lipofectamine 2000 have not been previously assessed in neuroblastoma cells (B104/B35), nor in primary AF or AE cultures. Transfection efficiencies obtained here (9–12%) for neuroblastoma and AE cultures are low compared to other mitotic cell types [2, 20], while transfection levels achieved here for primary cortical cultures were within the reported range [2, 11, 17]. Achieving high transfection rates is crucial for molecular manipulations such as gene silencing to attain maximal gene expression inhibition, or for quantitative and biochemical applications requiring high yield of the transgene product (mRNA or protein). Low transfection rate, on the other hand, is also desired as it offers the advantage of using neighboring un-transfected neurons as internal controls. This is ideal for applications evaluating neuronal phenotype, survival or protein localization in transfected vs naïve cells. It is also preferred in live-cell imaging experiments studying synaptic vesicle trafficking and endosomal dynamics [21]. Knowing the transfection rates that could be achieved using a certain method and how to modify it to target a particular cell type (in the context of heterogeneous cultures), are two important aspects when considering the suitability of a given transfection method for the application of interest. The results reported here can offer guidance for researchers with this regard. However, optimal transfection conditions and efficiency using the method reported here may need to be empirically determined when using other cell types.

## Additional file

**Additional file 1: Fig S1.** Effect of Lipofection on cell viability. **Fig S2.** Cell viability of transfected and control AE culture. **Fig S3.** Phenotyping of primary cortical cultures. **Fig S4.** Neuron- or astrocyte-specific transfection in primary mouse cortical cultures. **Fig S5.** Correlation between total number of cells and the number of transfected cells.

## Abbreviations

AC: astrocyte-containing culture
AF: astrocyte-free culture
AE: astrocytes enriched culture
DIV: days in vitro
hpt: hours post-transfection
