# Supplementary material for: Estimating transfection efficiency in differentiated and undifferentiated neural cells
Source: BMC Res Notes. 2019 Apr 15;12:225. doi: 10.1186/s13104-019-4249-5 (PMC6466792; doi:10.1186/s13104-019-4249-5)
Supplement: Supplementary file 1 — Additional file 1: Fig S1. Effect of Lipofection on cell viability. Fig S2. Cell viability of transfected and control AE culture. Fig S3. Phenotyping of primary cortical cultures. Fig S4. Neuron- or astrocyte-specific transfection in primary mouse cortical cultures. Fig S5. Correlation between total number of cells and the number of transfected cells. [file 13104_2019_4249_MOESM1_ESM.docx]

**Estimating transfection efficiency in differentiated and undifferentiated neural cells**

**Additional information**

**Materials and methods**

**Preparation of primary cortical neuronal and astrocyte cultures**

Cortical neurons were cultured from embryonic day 17.5 or 18.5 (E17.5/18.5) CD1 or C57BL/6 mouse pups as previously described [[1](#_ENREF_1)]. Dissociated cortices were plated at a density of 13 x 10^4^ cells/cm^2^ to achieve a final seeding of 250,000 cells per well in a 24-well plate. Three types of cortical cultures were generated: astrocyte-containing (AC), astrocyte-free (AF) and astrocytes enriched cultures (AE). To control for non-neuronal cell proliferation, cultures were grown in the presence or absence of the anti-mitotic drug Cytosine β-D-Arabinofuranoside hydrochloride (AraC). To obtain AC cultures, cells were treated with 9.6 µM/ml of AraC on 4 days *in vitro*-(DIV4). For AF preparations, two doses of 4.8 µM/ml AraC were applied. The first dose was added on DIV0 and the second on DIV4. AE cultures were prepared as previously described [[1](#_ENREF_1)], and grown in Dulbecco’s Modified Eagle Medium with GlutaMAX (DMEM; Gibco BRL, France) supplemented with 10% fetal bovine serum (FBS) and 1% penicillin-streptomycin (Gibco, France) in the absence of AraC. All experiments on AE preparations were performed in non-passaged primary cultures.

**Neuroblastoma cell lines culture**

Undifferentiated rat B35 and B104 neuroblastoma cells were both maintained at 37°C in a humidified incubator in 90% air and 10% CO_2_ atmosphere. Cells were routinely grown in 25cm^2^ flasks and subcultured when confluent. B35 cells were maintained in RPMI (Gibco, France) supplemented with 10% FBS and 1% penicillin-streptomycin (Gibco, France). B104 cells were grown in Dulbecco’s Modified Eagle Medium with GlutaMAX (DMEM; Gibco BRL, France) supplemented with 10% FBS and 1% penicillin-streptomycin. For subsequent experiments, cells were cultured in 6-well plates and maintained in 3-4 ml of growth media until the time of the experiment.

**Cell viability assessment**

Cell viability was determined using FITC Annexin V/Dead Cell Apoptosis Kit with FITC annexin V and PI, for Flow Cytometry (Invitrogen) according to the manufacture’s protocol. Since we are only interested in estimating cell death, regardless of the underlying process (apoptosis vs necrosis) or death stage (early vs late apoptosis), we only applied the red-fluorescent propidium iodide (PI) nucleic acid binding dye to distinguish between dead and live cells. This dye only permeates dead cells and is excluded from live cells. Briefly, harvested cells were washed 2X in cold 1X phosphate-buffered saline (PBS) then once with 1X annexin-binding buffer, before re-suspending them at a final concentration of ~1 x 10^6^ cells/ml in 100 µl of fresh 1X annexin-binding buffer. Cells were then transferred into a polypropylene tube for PI staining at final concentration 2µg/ml followed by 10 minutes incubation at room temperature. At the end of the incubation period, 400 µl of 1X annexin-binding buffer was gently mixed with the cells and incubated on ice for subsequent flow cytometry analysis.

PI staining was detected using BD FACSCalibur^TM^ Flow Cytometer (BD Biosciences) and analysis was performed with BD CellQuest^TM^  Pro Software (BD Biosciences). Gating was carried out using two-parameter density plots with forward scatter (cell size estimation) on the x-axis and PI stain on the y-axis (dead cells). Doublets and debris were excluded from the analysis. Cell viability at 48 hpt was represented as % dead/live cells based on PI stain and was compared to non-transfected control cells.

Additional cell viability assessment was performed only in AE cultures using nuclear staining with 4-,6-diamidino-2-phenylindole (DAPI). Cells were fixed and stained (as described in main text materials and methods section). Nuclear morphology was examined using Nikon epi-fluorescent microscope under a 10x objective lens, whereby cells with pyknotic or deformed nuclei were considered dead. Nuclei clusters and nuclear fragments were eliminated from the analysis. The number of dead/live cells was calculated as a percentage of the total cell population.

Results

Determining viability of transfected cells

In AE, relatively high toxicity was observed even in non-transfected cells despite of their normal morphological appearance (Fig. S1). The overestimation of dead cells may be due to false positive events resulting from PI staining of RNA (PI binding capacity is not exclusive to DNA, as it can intercalate into any double-stranded nucleic acid within the cell). This is a common occurrence particularly in cells with low nuclear:cytoplasmic ratios (>0.5) [[2](#_ENREF_2)]. While direct quantitation of nuclear:cytoplasmic ratios (NCR) in glia/astrocytes are not available, a rough estimate of (0.16) (based on a cell area of 74 µm and nuclear area of 10.50 µm [[3](#_ENREF_3), [4](#_ENREF_4)]) could be inferred when NCR is calculated as; [nuclear area: (cell area - nuclear area)] [[5](#_ENREF_5)].

Determining transfection efficiency in primary neurons

In an attempt to improve transfection efficiency, we trialed various Lipofectamine : DNA ratios in AC cultures and analyzed the cells 48 hpt. However, none of the tested ratios gave better results (Table S2). Moreover, Ohki and colleagues reported achieving a transfection rate up to 30% and 25% for primary hippocampal and cortical neurons, respectively. These levels were obtained using the same transfection reagent utilized here (Lipofectamine 2000), however, the transfection was performed on DIV4 instead of DIV7/8 [[6](#_ENREF_6)]. For this reason, we wanted to test if DIV4 would be the optimal time for transfection in our AC cultures. No improvement was observed; in fact transfection on DIV4 gave poorer results (Table S2). Possible sources of discrepancy include: 1) type of plasmid used (EGFP versus β-gal), although both are under the control of CMV-promoter, 2) differences in the transfection protocol, or 3) number of biological and technical repeats from which the data was derived (not clearly indicated by Ohki et al). Now the question arises on whether the plasmid used here gives sub-optimal results with primary neurons. To address this, we assessed the transfection efficiency using a different plasmid (pmaxGFP, 3486bp, Amaxa) in AC at 48 hpt. Similar results were obtained (Table S2).

Keeping in mind that variation between replicates is inherent to any biological system, a closer inspection of our data revealed a noticeable variability in the transfection efficiencies among the biological replicates. In addition, unlike transfected mitotic cells, we have noticed that the number of transfected primary neurons does not increase or decrease in proportion to the total number of cells imaged in each well (Tables S3 and S4). The protocol applied here appears to transfect (13-100) and (47-140) neurons/well, at 24 hpt and 48 hpt, respectively in AC cultures (Table S3). As for AF cultures, a range of (33-52) and (23-39) positive neurons/well was obtained at 24 hpt and 48 hpt, respectively (Table S4).

We therefore questioned if calculating the percentage of GFP^+^ cells can accurately reflect the transfection efficiency in these post-mitotic cells. To address this, we plotted the total number of cells (x-axis) versus the number of GFP^+^ ones (y-axis) for each culture preparation in order to analyze the relationship between these two variables by calculating Pearson correlation coefficients (*r^2^*) (Fig. S5). Both AC and AF cultures had the lowest coefficient values suggesting a poor correlation. On the other hand, good correlations were observed for the dividing cells studied herein. We do not know why the number of GFP^+^ neurons correlates poorly with the total number of cells. One possible explanation is variability in transgene expression among transfected primary neurons, whereby those producing smaller amounts of GFP (below the detection threshold of the fluorescence microscope) can be overlooked resulting in counting errors. In this case, other more sensitive methods could be employed to evaluate transfection efficiency such as flow cytometry [[7](#_ENREF_7), [8](#_ENREF_8)]. In addition to quantifying the percentage of reporter gene-expressing cells, amount of DNA uptake or reporter protein expression can be used as alternative readouts of transfection efficiency.

**Table S1. List of Antibodies used in this study.**

| **Primary antibody**  (dilution) | **Cat#/supplier** | **Secondary antibody** | **Cat# (**Jackson Immuno Research) |
| --- | --- | --- | --- |
| Anti-NeuN (1:400) | ABN78/ EMD Millipore | Biotin-SP Goat Anti-Rabbit | 111-066-144 |
| Anti-MAP2 (1:100) | M4403/SIGMA | Biotin-SP Goat Anti-Mouse | 115-065-164 |
| Anti-GFAP (1:500) | G3893/SIGMA | Cy™3 AffiniPure Goat Anti-Mouse | 115-165-146 |
| Anti-GFP (1:1000) | Ab290/abcam | Cy™2 AffiniPure Goat Anti-Rabbit | 111-165-144 |

**Table S2. Additional transfection conditions tested in this study.**

|  |  |  |  |  |
| --- | --- | --- | --- | --- |
|  | **Set#** | **Well#** | **DAPI^+^** | **GFP^+^/MAP2^+^** |
|  | Lipofectamine (µl): DNA (µg) 3.5:2 | | | |
| **transfection on DIV8** | 1 | B1 | 2456 | 58 |
|  | 1 | B2 | 2481 | 35 |
|  | 2 | B1 | 4858 | 26 |
|  | mean (%GFP^+^/MAP2^+^) =1.2 ± 0.6% | | | |
|  | 7:1 | | | |
|  | 1 | A1 | 1880 | 47 |
|  | 1 | A2 | 2740 | 34 |
|  | 2 | A1 | 4952 | 58 |
|  | mean (%GFP^+^/MAP2^+^) =1.5 ± 0.3% | | | |
|  | 7:2 | | | |
|  | 1 | A4 | 1821 | 40 |
|  | 1 | A5 | 2474 | 65 |
|  | 2 | A4 | 4460 | 21 |
|  | 2 | A5 | 5318 | 21 |
|  | mean (%GFP^+^/MAP2^+^) =1.4 ± 1.0% | | | |
|  | 3.5:1 (pmaxGFP) | | | |
|  | 1 | B5 | 4366 | 37 |
|  | 1 | B6 | 4223 | 40 |
|  | 2 | D5 | 3523 | 23 |
|  | 2 | D6 | 4561 | 19 |
|  | 3 | B1 | 4303 | 61 |
|  | 3 | B2 | 5120 | 28 |
|  | 3 | B3 | 4683 | 36 |
|  | 4 | D1 | 2756 | 31 |
|  | 4 | D2 | 4049 | 40 |
|  | 4 | D3 | 2826 | 43 |
|  | mean (%GFP^+^/MAP2^+^) =0.9 ± 0.1% | | | |
|  | 3.5:1 | | | |
| **transfection on DIV4** | 1 | B1 | 2453 | 22 |
|  | 1 | B2 | 2022 | 24 |
|  | 2 | D1 | 2876 | 20 |
|  | 2 | D2 | 2350 | 18 |
|  | 2 | D3 | 1652 | 14 |
|  | 3 | B1 | 4484 | 18 |
|  | 3 | B2 | 3751 | 28 |
|  | 3 | B3 | 4206 | 20 |
|  | 4 | D2 | 3242 | 28 |
|  | 4 | D3 | 3376 | 40 |
|  | mean (%GFP^+^/MAP2^+^) =1.0 ± 0.1% | | | |

Analysis was carried out in AC cultures transfected with pEGFP-N3 for 48h unless otherwise indicated. Mean ± S.E.M. Set: represents biological replicates from independent cultures. Well: represents technical replicates. DAPI^+^: grand total of cells in each well was determined as the sum of nuclei counted from all imaged fields (per well). GFP^+^/MAP2^+^: grand total of transfected neurons in each well was determined as the sum of GFP^+^/MAP2^+^  cells counted from all imaged fields (per well).

**Table S3. Counts of GFP^+^ neurons in AC cultures transfected on DIV7/8.**

| **Set#** | **Well#** | **DAPI^+^** | **GFP^+^/MAP2^+^** |
| --- | --- | --- | --- |
| *24 hpt* | | | |
| 1 | B1 | 2882 | 34 |
| 1 | B2 | 2159 | 21 |
| 1 | B3 | 2774 | 29 |
| 2 | B1 | 2394 | 26 |
| 2 | B2 | 1126 | 13 |
| 2 | B3 | 856 | 13 |
| 3 | C1 | 3770 | 20 |
| 3 | C2 | 3654 | 15 |
| 4 | B1 | 1679 | 44 |
| 4 | B2 | 1745 | 42 |
| 5 | D1 | 4281 | 47 |
| 5 | D2 | 3502 | 30 |
| 6 | B1 | 3675 | 100 |
| 6 | B2 | 3415 | 79 |
| 6 | B3 | 2851 | 69 |
| 7 | B1 | 6753 | 48 |
| 7 | B2 | 7029 | 31 |
| 7 | B3 | 6232 | 36 |
| *48 hpt* | | | |
| 1 | B1 | 3801 | 140 |
| 1 | B2 | 2622 | 88 |
| 1 | B3 | 2682 | 83 |
| 2 | D1 | 3665 | 79 |
| 2 | D2 | 2388 | 62 |
| 2 | D3 | 3586 | 75 |
| 3 | C1 | 2873 | 59 |
| 3 | C2 | 2291 | 47 |

Set: represents biological replicates from independent cultures transfected with pEGFP-N3. Well: represents technical replicates. DAPI^+^: grand total of cells in each well was determined as the sum of nuclei counted from all imaged fields (per well). GFP^+^/MAP2^+^: grand total of transfected neurons in each well was determined as the sum of GFP^+^/MAP2^+^  cells counted from all imaged fields (per well).

**Table S4. Counts of GFP^+^ neurons in AF cultures transfected on DIV7/8.**

| **Set#** | **Well#** | **DAPI^+^** | **GFP^+^/MAP2^+^** |
| --- | --- | --- | --- |
| *24 hpt* | | | |
| 1 | B4 | 3539 | 47 |
| 1 | B5 | 2558 | 33 |
| 2 | A1 | 425 | 39 |
| 2 | A2 | 496 | 48 |
| 2 | A3 | 430 | 40 |
| 3 | C1 | 1269 | 45 |
| 3 | C2 | 1073 | 41 |
| 4 | A1 | 610 | 34 |
| 4 | A2 | 568 | 43 |
| 4 | A3 | 496 | 43 |
| 5 | A1 | 1448 | 38 |
| 5 | A2 | 1676 | 52 |
| 5 | A3 | 1570 | 46 |
| *48 hpt* | | | |
| 1 | A1 | 443 | 31 |
| 1 | A2 | 713 | 30 |
| 1 | A3 | 741 | 39 |
| 2 | C1 | 872 | 37 |
| 2 | C2 | 851 | 36 |
| 2 | C3 | 960 | 34 |
| 3 | D1 | 221 | 25 |
| 3 | D3 | 255 | 23 |

Set: represents biological replicates from independent cultures transfected with pEGFP-N3. Well: represents technical replicates. DAPI^+^: grand total of cells in each well was determined as the sum of nuclei counted from all imaged fields (per well). GFP^+^/MAP2^+^: grand total of transfected neurons in each well was determined as the sum of GFP^+^/MAP2^+^  cells counted from all imaged fields (per well).


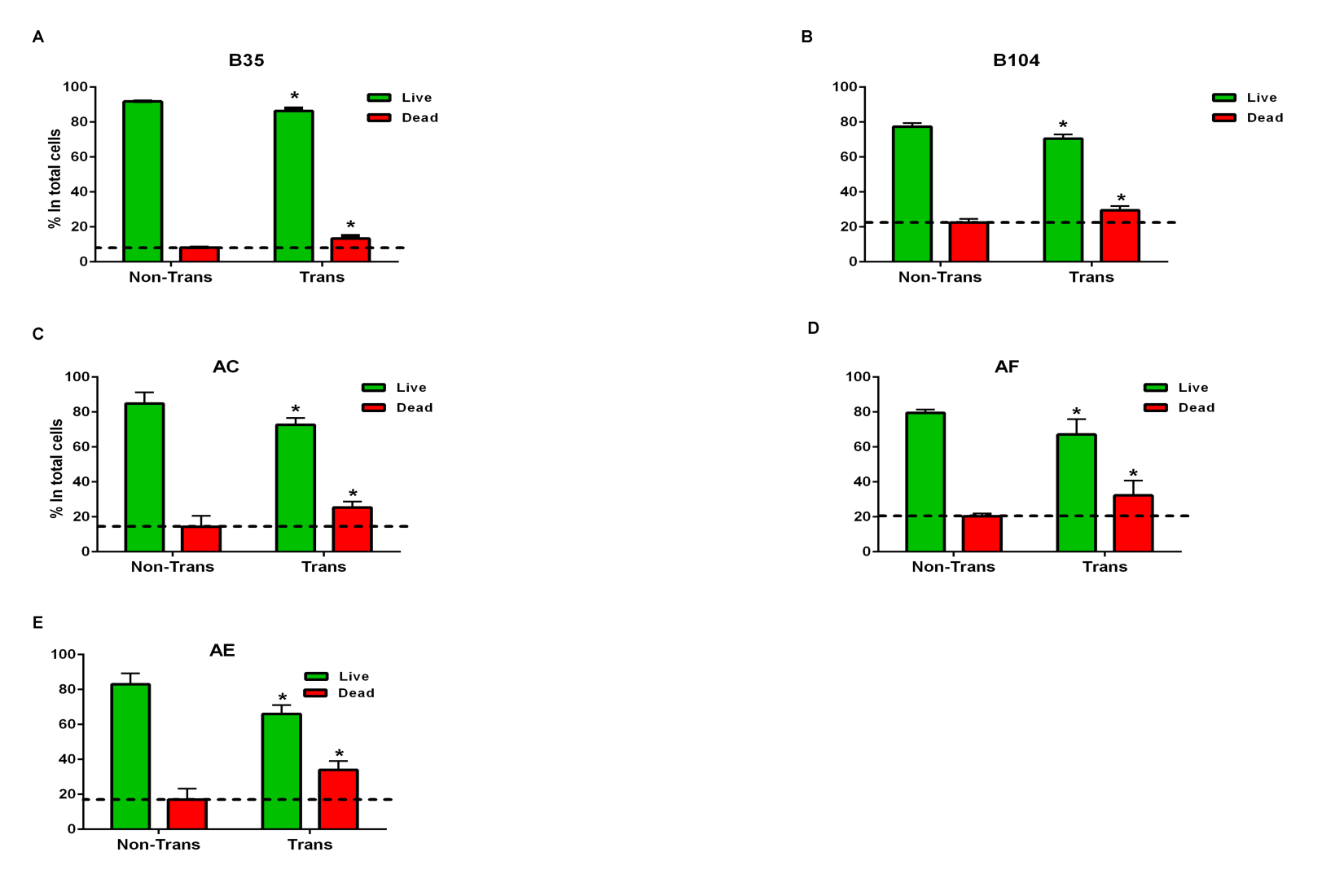


Fig. S1

Effect of Lipofection on cell viability. Cell viability experiments were carried out 48 hpt to allow for high transgene expression [[9](#_ENREF_9)] and estimation of toxicity at longer time points (48 vs 24 h). (A-D) Viability was assessed using PI staining and flow cytometry analysis on cells harvested 48 hpt, each experiment was done in duplicates (2 samples/condition) from 3 independent cultures. (E) Viability was determined based on nuclear morphology assessment using DAPI staining. Each AE experiment was done in replicates (2 or 3 wells/condition) from 4 independent cultures. Total number of cells quantified in each AE experiment was; (2089-10000 cells) for control (non-transfected) and (3280-5050 cells) for transfected cells. Data represents mean value ± S.E.M. ^*^*P*<0.01, Trans vs Non-trans, student T-test. (A) Non-Trans; live cells (mean value 91.8% S.E.M ± 0.1), dead cells (mean value 8.1% S.E.M ± 0.12) and Trans; live cells (mean value 86.3% S.E.M ± 0.9), dead cells (mean value 13.3% S.E.M ± 0.9). (B) Non-Trans; live cells (mean value 77.4% S.E.M ± 1.2), dead cells (mean value 22.5% S.E.M ± 1.2) and Trans; live cells (mean value 70.5% S.E.M ± 1.4), dead cells (mean value 29.5% S.E.M ± 1.4). (C) Non-Trans; live cells (mean value 84.8% S.E.M ± 3.8), dead cells (mean value 14.4% S.E.M ± 3.6) and Trans; live cells (mean value 72.7% S.E.M ± 2.2), dead cells (mean value 25.4% S.E.M ± 1.9). (D) Non-Trans; live cells (mean value 79.5% S.E.M ± 1.1), dead cells (mean value 20.3% S.E.M ± 0.9) and Trans; live cells (mean value 67.1% S.E.M ± 5.4), dead cells (mean value 32.3% S.E.M ± 4.9). (E) Non-Trans; live cells (mean value 83% S.E.M ± 3.1), dead cells (mean value 17% S.E.M ± 3.1) and Trans; live cells (mean value 66% S.E.M ± 2.7), dead cells (mean value 34% S.E.M ± 2.5). Non-Trans: non-transfected controls. Trans: Transfected cells.


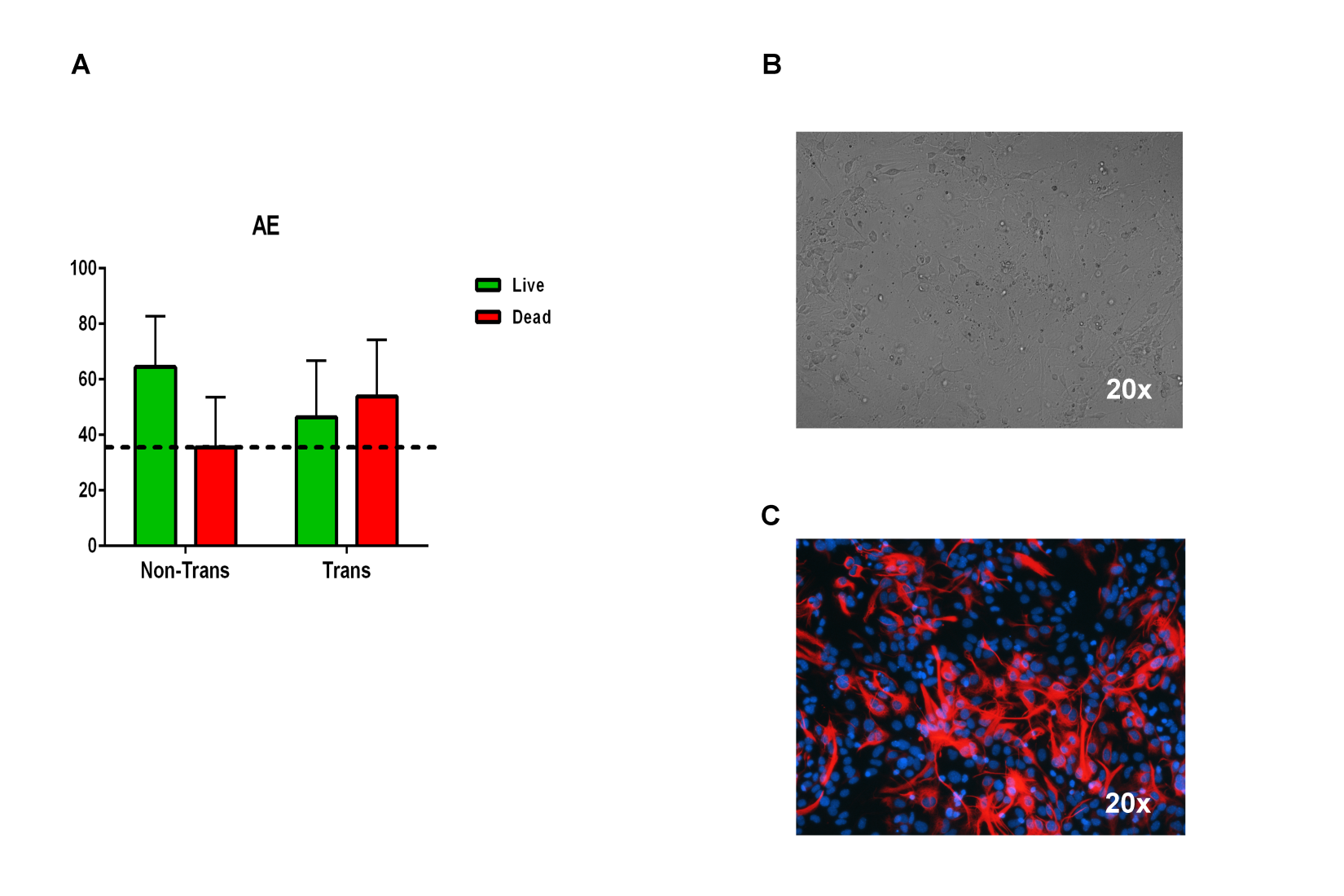


**Fig. S2**

**Cell viability of transfected and control AE culture.** (A) Viability was assessed using PI staining and flow cytometry analysis on cells harvested 48 hpt, each experiment was done in duplicates (2 samples/condition) from 3 independent cultures. Data represents mean value ± S.E.M. ^*^*P*<0.01, Trans vs Non-trans, student T-test. Non-Trans; live cells (mean value 64.5% S.E.M ± 9.2), dead cells (mean value 35.5% S.E.M ± 9.0) and Trans; live cells (mean value 46.3% S.E.M ± 10.2), dead cells (mean value 53.8% S.E.M ± 10.2). (B,C) Non-trans AE culture on DIV8. (B) Bright field images of cells exhibiting classical morphological features of healthy type-1 astrocytes resembling fibroblasts [[10](#_ENREF_10)]. (C) The identity and health of these cells was confirmed by the positive GFAP staining and normal appearance of DAPI stained nuclei.


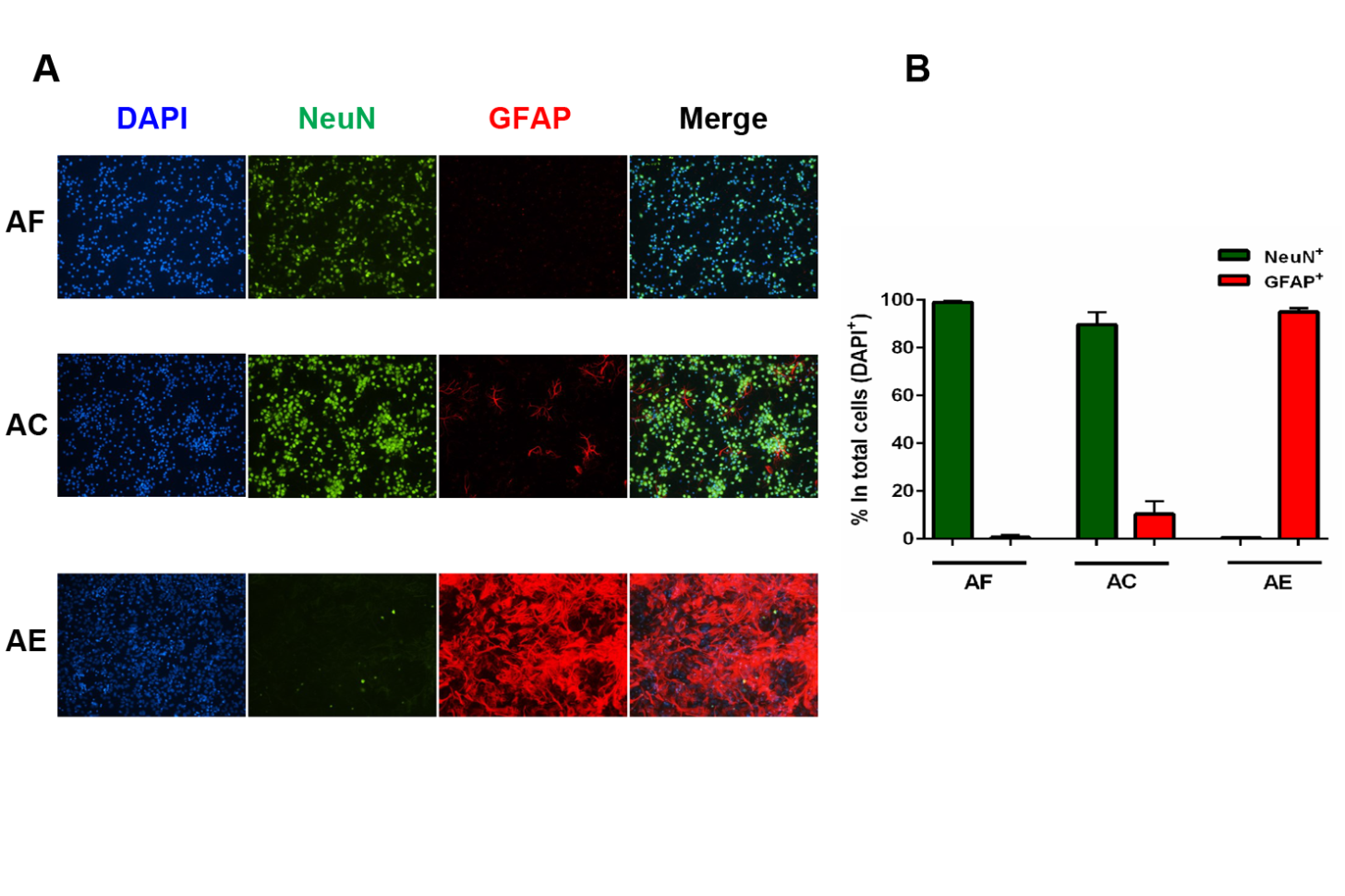


**Fig. S3**

**Phenotyping of primary cortical cultures.** (A) Representative images of AF, AC and AE cultures. About 7-9 fields were imaged per well and each experiment was done in triplicate (3 wells/condition) from 4 or 6 independent cultures. Total number of cells quantified in each experiment was; (1500-6400 cells) for AC, (430-6500 cells) for AF and (6300-9950) for AE. (B) Quantitative analysis of the percentage of NeuN^+^ (neurons, green) and GFAP^+^ (astrocytes, red) among the total DAPI^+^ cells (total population, blue). Data represents mean value ± S.E.M. AF cultures consisted of 99.06% ± 0.38 NeuN^+^ neurons, AC cultures were composed of 90.52% ± 1.23 NeuN^+^ neurons and 9.47% ± 1.23 GFAP^+^ astrocytes, and AE cultures were composed of 94.91% ± 0.82 GFAP^+^ astrocytes.

**
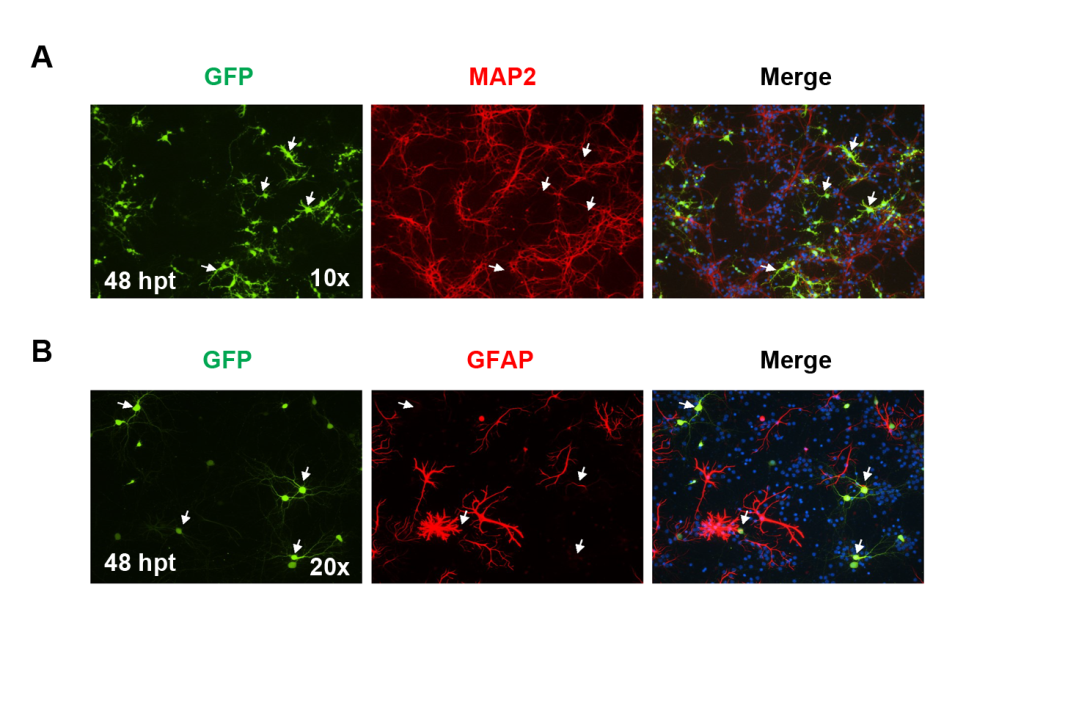
**

**Fig. S4**

**Neuron- or astrocyte-specific transfection in primary mouse cortical cultures.** Immunofluorescence analysis in AC cultures 48 hpt. (A) Cultures transfected at DIV2 contain mostly GFP^+^ astrocytes as evident from the typical star-like morphology and negative MAP2 staining. (B) Cultures transfected at DIV7/8 contain mostly GFP^+^ neurons as evident from the typical neuron morphological features such as pyramidal somas and long extended extensions, in addition to negative GFAP staining.


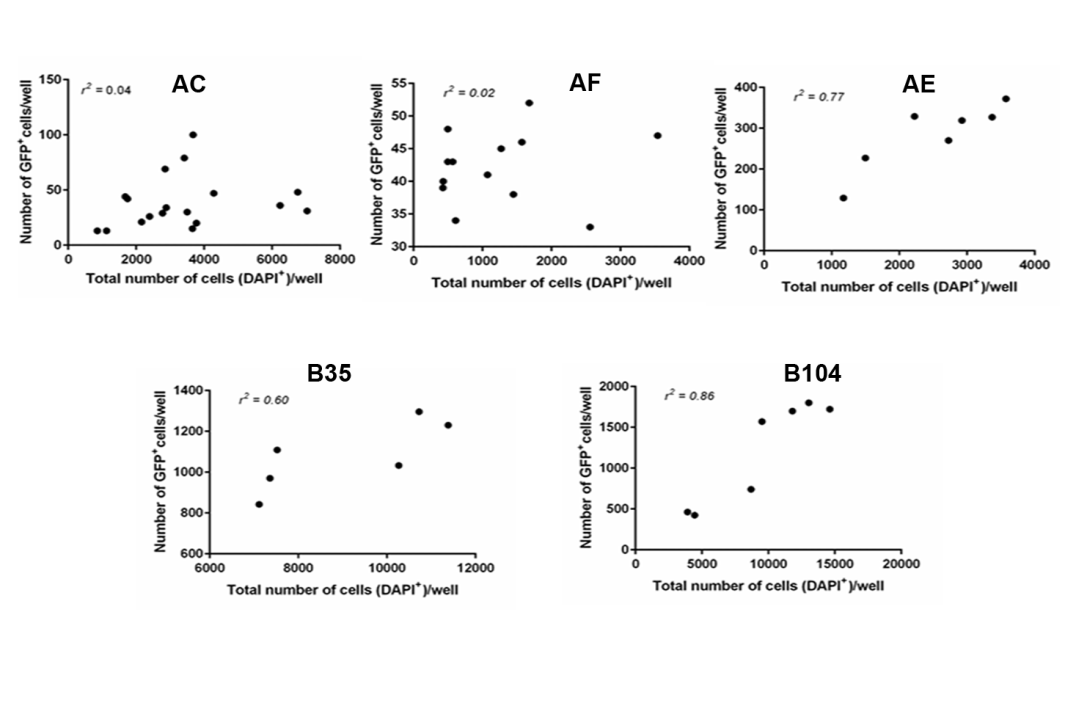


**Fig. S5**

**Correlation between total number of cells and the number of transfected cells.** The relationship between the total number of cells (DAPI^+^) and the number of transfected (GFP^+^) cells/well was assessed for each culture preparation 24 hpt. Analysis was performed in GraphPad Prism 6. r^2^= Pearson correlation coefficient value.

**References**

1. Bell KF, Al-Mubarak B, Martel MA, McKay S, Wheelan N, Hasel P, et al. Neuronal development is promoted by weakened intrinsic antioxidant defences due to epigenetic repression of Nrf2. Nat Commun. 2015;6:7066. doi: 10.1038/ncomms8066. PubMed PMID: 25967870

2. Rieger AM, Hall BE, Luong le T, Schang LM, Barreda DR. Conventional apoptosis assays using propidium iodide generate a significant number of false positives that prevent accurate assessment of cell death. J Immunol Methods. 2010;358(1-2):81-92. doi: 10.1016/j.jim.2010.03.019. PubMed PMID: 20381494

3. Fedoroff S, McAuley WA, Houle JD, Devon RM. Astrocyte cell lineage. V. Similarity of astrocytes that form in the presence of dBcAMP in cultures to reactive astrocytes in vivo. J Neurosci Res. 1984;12(1):14-27. doi: 10.1002/jnr.490120103. PubMed PMID: 6090683

4. Haseleu J, Anlauf E, Blaess S, Endl E, Derouiche A. Studying subcellular detail in fixed astrocytes: dissociation of morphologically intact glial cells (DIMIGs). Front Cell Neurosci. 2013;7:54. doi: 10.3389/fncel.2013.00054. PubMed PMID: 23653590

5. Teryukova NP, Sakhenberg EI, Ivanov VA, Snopov SA. Establishment and characterization of clonal lines with cancer stem- and progenitor-cell properties from monolayer Zajdela hepatoma. Cell and Tissue Biology. 2017;11(2):161-71. doi: 10.1134/S1990519X17020079.

6. Ohki EC, Tilkins ML, Ciccarone VC, Price PJ. Improving the transfection efficiency of post-mitotic neurons. J Neurosci Methods. 2001;112(2):95-9. PubMed PMID: 11716945

7. Homann S, Hofmann C, Gorin AM, Nguyen HCX, Huynh D, Hamid P, et al. A novel rapid and reproducible flow cytometric method for optimization of transfection efficiency in cells. PLoS One. 2017;12(9):e0182941. doi: 10.1371/journal.pone.0182941. PubMed PMID: 28863132

8. Marjanovic I, Kanduser M, Miklavcic D, Keber MM, Pavlin M. Comparison of flow cytometry, fluorescence microscopy and spectrofluorometry for analysis of gene electrotransfer efficiency. J Membr Biol. 2014;247(12):1259-67. doi: 10.1007/s00232-014-9714-4. PubMed PMID: 25146882

9. Sariyer IK. Transfection of neuronal cultures. Methods Mol Biol. 2013;1078:133-9. doi: 10.1007/978-1-62703-640-5_11. PubMed PMID: 23975826

10. Raff MC, Abney ER, Cohen J, Lindsay R, Noble M. Two types of astrocytes in cultures of developing rat white matter: differences in morphology, surface gangliosides, and growth characteristics. J Neurosci. 1983;3(6):1289-300. PubMed PMID: 6343560
